# Semantic fluency predicts survival of memory clinic patients

**DOI:** 10.1186/s13195-026-02152-y

**Published:** 2026-08-07

**Authors:** Lars Frings, Joachim Brumberg, Philipp T. Meyer

**Affiliations:** Department of Nuclear Medicine, Medical Center - Faculty of Medicine, University of Freiburg, Hugstetter Str. 55, Freiburg, 79106 Germany

**Keywords:** Dementia, Alzheimer’s Disease, Cognition, Survival, Mortality, Amyloid PET, FDG PET

## Abstract

**Background:**

Semantic fluency is commonly assessed in the diagnostic work-up of individuals with suspected cognitive impairment due to neurodegenerative disease. Semantic fluency has a predictive value for survival in clinical AD and in healthy elderly individuals, but it is unknown if this also applies to biomarker-confirmed AD and non-AD memory clinic patients. Potential associations with and added value of imaging biomarkers of amyloid pathology and neurodegeneration have yet to be explored.

**Methods:**

From our clinical registry, we included patients who were assessed at a memory clinic with the neuropsychological assessment battery of the Consortium to Establish a Registry for Alzheimer’s Disease (CERAD-NAB) and whose vital status could be retrieved in 07/2024.

We tested the association of semantic fluency performance at first assessment and over time (and, for comparison, further CERAD-NAB subtest scores) with mortality risk using age-adjusted single-predictor Cox proportional hazard models. Amyloid status (positive vs negative on clinical PET reads) and global cognitive impairment (MMSE) were included as covariates.

In addition, we explored associations between semantic fluency performance and both regional cortical glucose metabolism (FDG PET) and global amyloid load (centiloids; PiB PET). Finally, the predictive value of global amyloid load and glucose metabolism in comparison to and in combination with semantic fluency performance was assessed.

**Results:**

583 patients were included (age 68.9 ± 8.7, 45% female). 280 patients (48%) had died and 303 (52%) were alive after a median of 8.0 years [95% C.I. 7.7—8.4].

Better semantic fluency (age-, sex- and education adjusted Z score, based on normative data) was significantly associated with lower mortality risk (HR = 0.71 [0.63 – 0.80], Bonferroni-corrected *p* < 0.001). Its predictive value was higher than that of all other CERAD-NAB subscores (e.g., memory, visuospatial abilities). Semantic fluency remained a significant predictor when amyloid status and global cognitive impairment were accounted for (HR = 0.75 [0.61 – 0.91], *p* = 0.0035). In patients with more than one assessment (*N* = 163), longitudinal change of semantic fluency (derived from a linear mixed effects model) was also a significant predictor (HR = 0.66 [0.53—0.83], *p* < 0.001).

In 218/583 patients who had received amyloid and FDG PET, worse semantic fluency was associated with decreased FDG uptake of left inferior and middle temporal, dorsolateral frontal, and posterior parietal cortical regions (Bonferroni-corrected *p* < 0.05), but not with amyloid load. FDG uptake of the left IFG (pars opercularis) was itself a predictor of survival (HR = 0.68 [0.55 – 0.84], Bonferroni-corrected *p* < 0.05), but to a lesser degree than semantic fluency (and MMSE, naming and figure drawing). Predictive accuracy of semantic fluency was further improved by including FDG uptake of the right anterior cingulate (Bonferroni-corrected *p* = 0.078). Global amyloid load was not associated with survival.

**Conclusions:**

Survival of memory clinic patients can be predicted by semantic fluency, independently from amyloid status and global cognitive impairment. Anterior cortical glucose metabolism is itself a significant predictor of survival and slightly improves prediction by semantic fluency.

**Supplementary Information:**

The online version contains supplementary material available at https://doi.org/10.1186/s13195-026-02152-y.

## Background

Diagnosis of memory clinic patients, particularly AD patients, has dramatically improved over the last decades, which is mainly due to biomarker developments. However, disease-defining imaging or liquid biomarkers of disease pathology (e.g., PET or cerebrospinal fluid) are not widely available, and clinical diagnosis alone is often incorrect, even in specialized settings [1]. Still, when counseling of patients and relatives addresses the prognosis, e.g., estimation of survival duration, this is often based on the clinically assumed diagnosis.

Neuropsychological assessment is recommended in dementia diagnostic criteria and is hence standard in memory clinics to aid the diagnostic decision. Scores from neuropsychological tests reflect (regional) brain health and disease severity. Thus, neuropsychological test scores might bear the potential to more directly infer estimates of survival duration/mortality risk than coarse diagnostic categories. A commonly applied test battery is the neuropsychological assessment battery of the Consortium to Establish a Registry for Alzheimer’s Disease (CERAD-NAB) [2], which includes tests of memory, but also semantic fluency.

Interestingly, one previous study showed that semantic fluency predicts survival in clinically diagnosed AD [3]. However, due to a lack of biomarker-confirmation of the clinical AD diagnosis, it is not clear whether the patient sample contained patients with neurodegenerative diseases other than AD, e.g., limbic-predominant age-related TDP-43 encephalopathy (LATE) or frontotemporal lobar degeneration (FTLD). Particularly the presence of FTLD patients in the patient sample might have biased the survival outcome on one side [4, 5] and assessment of semantic fluency on the other [6]. Intriguingly, in a very recent study Ghisletta et al. [7] demonstrated that semantic fluency predicts survival also in healthy elderly individuals. The previous studies that demonstrated the predictive value of semantic fluency alone [7] or semantic fluency combined with phonemic fluency [3] used different, large sets of neuropsychological tests covering a wide range of cognitive domains (Cosentino et al.,: verbal fluency, memory, abstract reasoning, visuospatial functioning, and language; Ghisletta et al.: verbal fluency, perceptual speed, episodic memory, verbal knowledge, and general intelligence). Importantly, among all these domains and tasks, both identified semantic fluency alone [7] or in a combined score with phonemic fluency [3] as the best predictors of survival. Impaired semantic fluency may reflect disruption of frontotemporal networks supporting semantic retrieval and executive search processes [8]. Frontal lobe dysfunction may, on one end of the spectrum, promote risk-taking behavior and poor medication adherence, or, conversely, lead to apathy and diminished self-initiated activity [9]. The latter may adversely affect musculoskeletal and cardiovascular health through prolonged immobility or bed rest [10, 11]. Both behavioral extremes may ultimately contribute to an increased risk of mortality.

Given its clinical importance, we explored whether semantic fluency performance has a predictive value for survival of memory clinic patients and, in particular, independently from amyloid status (on amyloid positron emission tomography (PET)) and global cognitive impairment. Moreover, we explored associations of semantic fluency with established imaging biomarkers of AD (i.e., amyloid PET and cerebral glucose metabolism on [^18^F]fluorodeoxyglucose (FDG) PET) and their possible added values.

## Methods

### Patients

Patients were retrospectively identified by searching the clinical registry of a tertiary referral center. All patients gave written informed consent to the retrospective analysis of clinical data. The study was performed in accordance with the ethical standards as laid down in the 1964 Declaration of Helsinki and its later amendments. The ethics committee of the Albert-Ludwigs-Universität Freiburg, Germany approved the study protocol (application No. 23–1276-S1-retro). We included cases who were referred for dementia diagnostic workup and (1) had a neuropsychological assessment at a memory clinic with the CERAD-NAB and (2) whose vital status could be retrieved in 07/2024 from the state resident registration. For a subgroup analysis (associations with amyloid and cerebral glucose metabolism), we included only those patients who additionally had received amyloid PET and FDG PET (from 05/2009 to 07/2024).

### Neuropsychological test scores

We evaluated clinical assessments and documentation of the CERAD-NAB in its German version [12] and included semantic fluency and ten more test scores from the original version of the CERAD-NAB. In detail, we analyzed the raw score of the Mini-Mental Status Examination (MMSE; included in the CERAD-NAB) and age-, sex-, and education-adjusted z-scores (indicating deviation from normative data) from the following CERAD-NAB subtest scores: semantic fluency (animals; 1 min time limit), naming, wordlist learning, wordlist recall, wordlist recall as proportion of items previously learned, wordlist recognition, wordlist intrusions, figure drawing, figure recall, figure recall as proportion of correctly drawn. Scores from subtests which were later appended to the so-called ‘CERAD-plus’ test battery (phonological fluency, Trail Making Test-A, and Trail Making Test-B) were not included, as they were not consistently used in the memory clinic.

### Biomarkers

Amyloid PET is a ‘core 1 biomarker’ for AD, and an amyloid PET scan read by an expert as ‘positive’ will nearly always coincide with intermediate-to-high AD neuropathologic change [13]. Patients with an amyloid PET visually read in clinical routine as ‘positive’ were hence regarded as having biomarker-confirmed AD. For clinical routine visual reads, static images were acquired 40—60 min after injection of 470 ± 99 (Siemens ECAT EXACT 922/47 PET system), 454 ± 60 (Philips Gemini TrueFlight 64 PET/CT system), or 485 ± 81 (Philips Vereos PET/CT system) MBq [^11^C]PIB. Clinical visual reads (dichotomized into A + or A-) and centiloids (CL; calculated with an in-house protocol, following GAAIN recommendations, https://www.gaain.org/centiloid-project) were used in the following analyses.

[^18^F]FDG PET data were acquired on a Siemens ECAT EXACT 922/47 PET system (40–60 min p.i.), Philips Gemini TrueFlight 64 PET/CT system (50–60 min p.i.), or Philips Vereos PET/CT system (50–60 min p.i.) after injection of 308 ± 29, 211 ± 8, or 222 ± 49 MBq [^18^F]FDG, respectively. Data were reconstructed following standard protocols and preprocessed with an in-house pipeline that uses parts of the SPM12 software and involves spatial normalization of the individual [^18^F]FDG PET to an in-house [^18^F]FDG PET template in MNI space and spatial filtering resulting in approximately 9 mm final isotropic resolution (applying Gaussian filters of 5, 7, or 8 mm FWHM for Siemens ECAT, Philips TF64, and Philips Vereos images, respectively), and scaling to the brain parenchyma. Normalized regional uptake values of 68 ROI of the Desikan-Killiany atlas [14] were extracted and used in the analyses. FDG PET was performed 58 days (median; IQR = 30–125), amyloid PET was performed 84 days (median; IQR = 49–196) after neuropsychological assessment.

### Statistics

We first tested the predictive value of the demographic variables age, sex, and years of education, in order to determine variables that needed to be accounted for in the following analyses.

1. We tested the predictive value of semantic fluency and, for comparison, of ten more subtest scores (naming; MMSE; wordlist learning, recall, recall as proportion of learnt items, intrusions, and recognition; figure drawing, figure recall, and figure recall as proportion of drawn items) in single-predictor Cox proportional hazard analyses (age-adjusted; with years after (first) neuropsychological assessment as time scale) and computed the magnitude of the Wald z-value (i.e., the absolute value of log(HR)/SE) as a standardized measure of effect strength. We compared the predictive value of semantic fluency (age-adjusted model) with that of age alone using an analysis of deviance. As a confirmatory analysis, a multivariable Cox proportional hazards model was estimated that included the eleven CERAD-NAB test scores as well as age. This was carried out in order to reveal significant predictor variables after correction for all other variables.
2. In order to test whether an association of semantic fluency with mortality was present independently from amyloid status and global cognitive impairment, we included amyloid status and MMSE score as covariates in two separate Cox proportional hazard models with semantic fluency as predictor (age-adjusted).
3. In patients who had at least two assessments of semantic fluency, longitudinal change of semantic fluency performance was calculated based on a linear mixed effects model with random intercepts and random slopes for follow-up time to quantify individual change in semantic fluency. Intercept–slope covariance was omitted to improve model stability. Individual change was extracted from the model fit and subsequently tested for its predictive value for survival in an age-adjusted Cox model.
4. In patients with available amyloid and FDG PET, we determined associations of (baseline) semantic fluency performance with normalized regional FDG uptake and global amyloid load (CL) by a set of Pearson correlation analyses. This was done in order to provide background information about the specific brain regions on which semantic fluency relies.
5. We determined the predictive value of normalized regional FDG uptake and global amyloid load (CL) (and the 11 CERAD-NAB subtest scores, for comparison in this PET subgroup) by a series of age-adjusted single-predictor Cox proportional hazards models.
6. We further explored whether the reference model (prediction by semantic fluency + age) improved, in terms of the likelihood ratio, by adding global amyloid load or FDG uptake to the reference model (per region; analysis of deviance and likelihood ratio test).

Statistical analyses were carried out in R language and environment for statistical computing version 4.5.0. We report results at a threshold for statistical significance of uncorrected *p* < 0.05 or, when series of separate univariate analyses were applied, Bonferroni-corrected *p* < 0.05.

## Results

We identified 583 patients (Table 1) who completed the CERAD-NAB without missing data in any of the eleven CERAD variables and retrievable vital status; 280 patients (48%) had died and 303 (52%) were alive after a median of 8.0 years [95% C.I. 7.7—8.4] as estimated by the reversed Kaplan–Meier method. Filed clinical diagnoses (available in *N* = 438 (75%) of our patients) were mild cognitive impairment (MCI; *N* = 164; 28%), AD (*N* = 122; 21%), frontotemporal dementia (FTD; *N* = 49; 8%), mixed dementia (*N* = 46; 8%), dementia with Lewy bodies (DLB; *N* = 31; 5%), depression (*N* = 15; 3%), and vascular dementia (*N* = 11; 2%). Of the 583 included patients, 218 additionally had received amyloid PET and FDG PET (please see below for analyses of this subgroup).

**Table 1:** Patient characteristics

	Total Group	Subset of patients with amyloid and FDG PET
N	583	218
Age (years; M ± S.D.)	68.9 ± 8.7	67.9 ± 8.5
Sex (m:f)	322:261 (55%:45%)	114:104 (52%:48%)
Education (years; M ± S.D.)	13.3 ± 3.5	13.3 ± 3.5
Amyloid PET (positive:negative)	-	132:86 (61%:39%)
Amyloid load (centiloids; mean ± S.D.)	-	55.7 ± 51.1
MMSE (points; M ± S.D.)	23.9 ± 3.5	23.4 ± 3.4

A Cox proportional hazard model including the predictor variables age, sex, and years of education revealed that only age was a significant predictor (HR = 1.05 [1.03—1.07], *p* < 0.001), but sex (HR_female_ = 0.82 [0.64—1.05], *p* = 0.119) and years of education (HR = 0.97 [0.94—1.01], *p* = 0.101) were not.

### Analyses of cognitive variables

For a tabulated overview of the following six analysis steps, please see Supplementary Table 1.

**Fig. 1:**
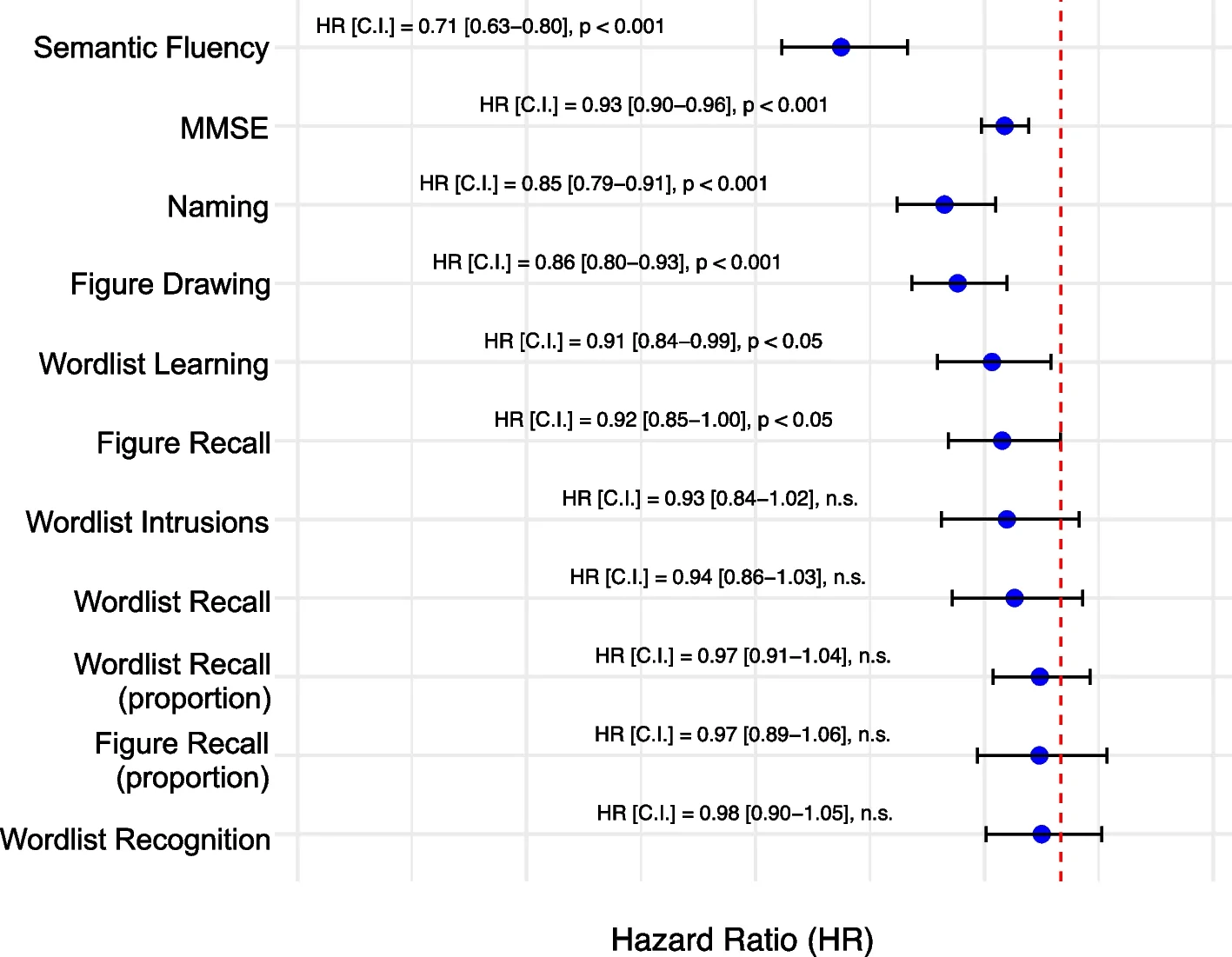
Hazard ratios from separate single-predictor Cox proportional hazard model estimations (age-adjusted) testing the predictive value of semantic fluency and other CERAD-NAB scores, in order of descending absolute values of the Wald z statistic (log(HR)/SE). C.I. refers to 95% confidence intervals

**Fig. 2:**
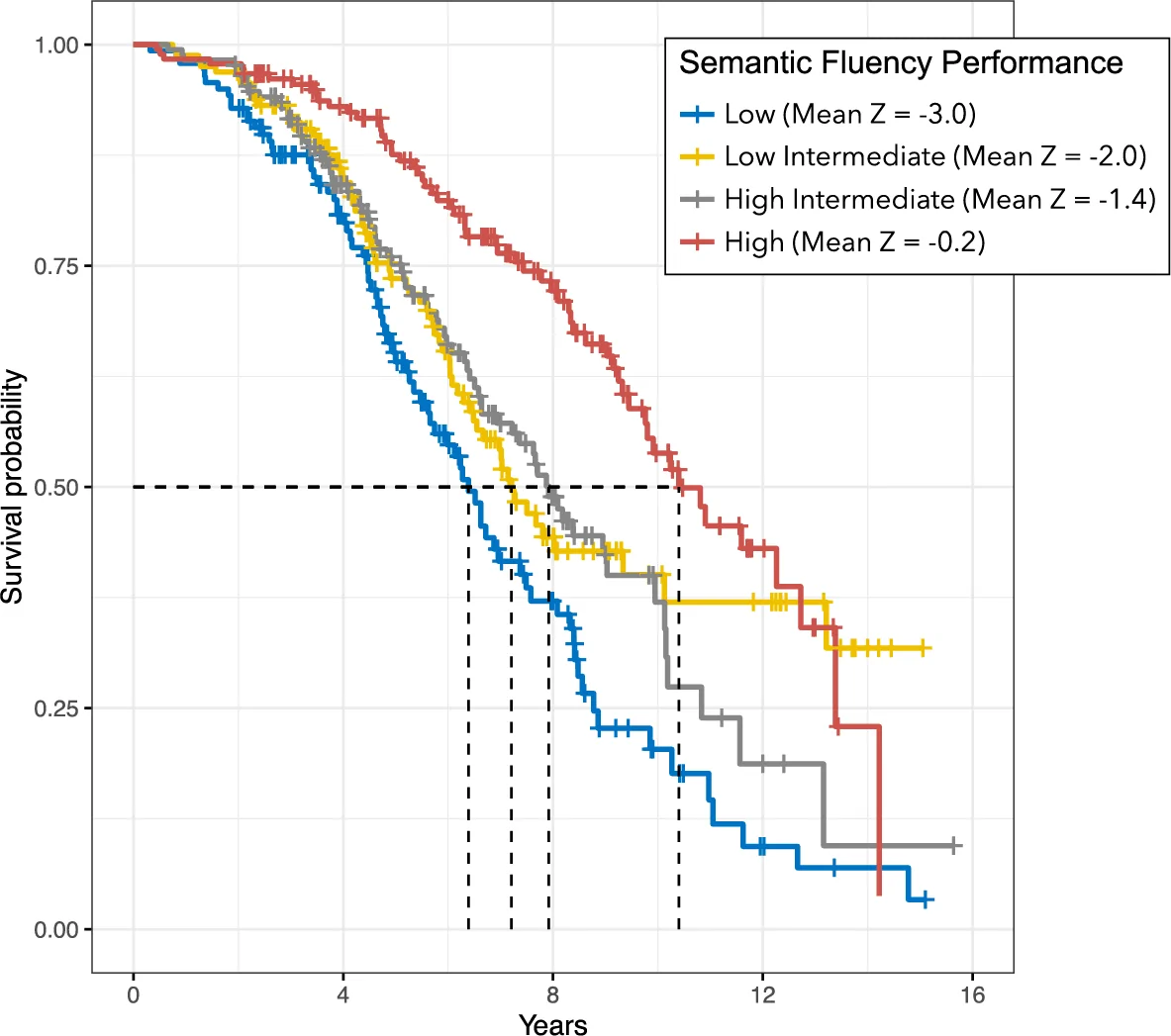
Patients with worse semantic fluency performance had shorter survival: age-adjusted survival curves of semantic fluency quartiles. Median survival for each group (from worst to best performance) was 6.39 [5.62—7.58], 7.21 [6.48—13.21], 7.92 [6.64—10.13], and 10.40 [9.45—not defined] years. (Quartile split of the continous variable for display purposes only)

1. A series of age-adjusted single-predictor Cox proportional hazard models revealed that the best predictor of survival was semantic fluency (age-adjusted, *N* = 583): HR = 0.71 [0.63—0.80], *p* < 0.001, Bonferroni-corrected for 11 tests. Hence, mortality risk increased by 40% per decrease of one unit in semantic fluency performance (age-, sex-, and education-adjusted z score). The absolute Wald z statistic for semantic fluency was 5.8; it was the highest among all eleven tests (see Fig. 1). As individual risk depends on both, semantic fluency and age, Table 2 provides mortality risk by combinations of both predictors, age and semantic fluency. Harrell’s c of the age-adjusted model was 0.67. Figure 2 depicts survival curves by semantic fluency performance split in quartiles (for display purposes only). Further subtest scores with a significant predictive value (age-adjusted) were MMSE, naming, figure drawing, wordlist learning, and figure recall (for detailed results, see Fig. 1 and Supplementary Table 1).

**Table 2:** Mortality risk** (**hazard ratio) at combinations of age and semantic fluency performance (age-, sex-, and education-adjusted Z-scores)

**Age**	**Semantic Fluency (z)**				
	**0**	**−1**	**−2**	**−3**	**−4**
50	1 (Reference)	1.4	2.0	2.8	3.9
60	1.9	2.6	3.7	5.1	7.2
70	3.4	4.8	6.8	9.5	13.4
80	6.4	8.9	12.6	17.6	24.7
90	11.8	16.6	23.3	32.7	45.9

The predictive value of semantic fluency and age combined was higher than that of age alone (chi^2^ = 34.6, df 1, uncorrected *p* < 0.001).

Best prediction by semantic fluency among the CERAD-NAB subtest scores was confirmed by the multivariable Cox proportional hazard model that identified semantic fluency as the only significant test score (HR = 0.73 [0.63—0.84], uncorrected *p* < 0.001; for details, see Supplementary Table 2). In order to account for collinearity between cognitive variables, we replicated the multivariable age-adjusted Cox model by using an elastic net Cox regression with all cognitive predictor variables. Results showed that semantic fluency retained predictive contribution after accounting for correlated variables, with an HR of 0.75 [bootstrap 95% C.I. 0.64–0.85]. The elastic net Cox model retained eight further variables besides age and semantic fluency: figure drawing (0.89 [0.77—1.00], MMSE (0.91 [0.74–1.00]), naming (0.92 [0.81–1.00]), wordlist intrusions (0.93 [0.81–1.00]), wordlist learning (1.10 [1.00–1.34]), wordlist recall (1.03 [1.00–1.32]), figure recall (proportion) (1.05 [0.96–1.49]), and wordlist recognition (1.10 [1.00–1.33]).

(2) Semantic fluency remained a significant predictor (HR = 0.75 [0.66–0.85] and HR = 0.70 [0.58–0.84], respectively, both uncorrected *p* < 0.001), when controlling for MMSE or amyloid status.
(3) Longitudinal change of semantic fluency performance

163/583 patients (28%; 69.2 ± 7.7 years of age; 45% female) had at least two assessments of semantic fluency; i.e., 402 assessments in total, over up to 10 years (minimum = 2, maximum = 11 assessments; numbers of patients with 2, 3, or more than 3 assessments: 121, 24, 18). 70/163 died in a median follow-up duration of 9.4 years [9.1–11.2]. Average annual semantic fluency change as derived from the linear mixed effects model was −0.12 ± 0.05 (negative value indicates decline) in Z scores adjusted for age, sex, and education. The age-adjusted Cox model revealed that longitudinal change of semantic fluency performance (z-transformed) significantly predicted survival: lesser decline was protective (HR = 0.66 [0.53–0.83], *p* < 0.001). The absolute Wald z statistic for longitudinal change of semantic fluency was 3.6; Harrell’s c of the age-adjusted model was 0.63. For comparison, longitudinal change was also computed for the remaining CERAD-NAB subtest scores and their predictive value for survival was tested. This revealed that the only other subtest score that significantly predicted survival was the MMSE (HR = 0.62 [0.52–0.76], *p* < 0.001, *n* = 202 (35%); please see Supplementary Table 3 for details).

### Subgroup analyses in patients with FDG PET and amyloid PET

**Fig. 3:**
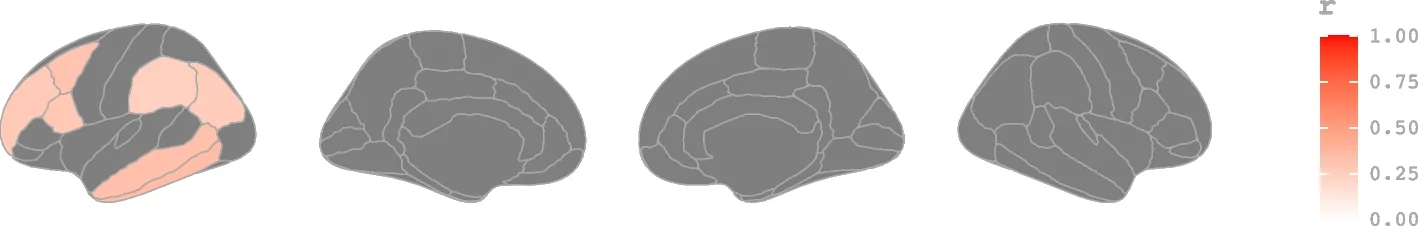
Semantic fluency was associated with decreased normalized FDG uptake in temporal, frontal, and parietal cortex of the left hemisphere (*p* < 0.05, Bonferroni-corrected). Left lateral, left medial, right medial, and right lateral views

**Fig. 4:**
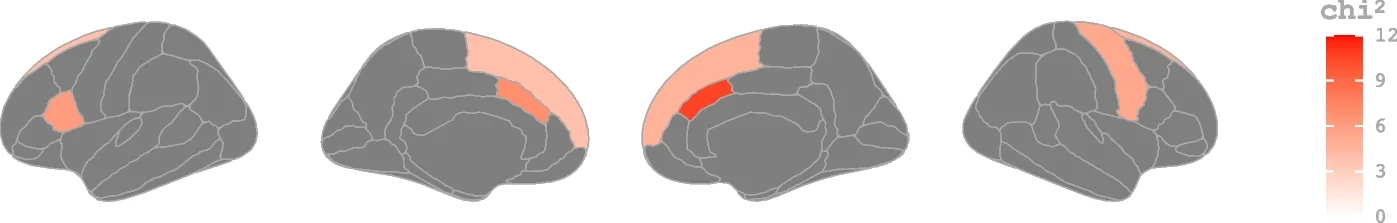
Cortical regions whose FDG uptake improved the prediction accuracy of the reference model (prediction of survival by semantic fluency + age); separate analyses of deviance, *p* < 0.05, uncorrected. Left lateral, left medial, right medial, and right lateral views

(4) In a subgroup of 218 patients with amyloid and FDG PET data, worse semantic fluency was associated with decreased normalized FDG uptake of left inferior and middle temporal, dorsolateral frontal, and posterior parietal cortical regions (Pearson r from 0.35 to 0.24, *p* < 0.05, Bonferroni-corrected for 69 tests, respectively; Fig. 3). Semantic fluency was not correlated with amyloid load (Pearson *r* = 0.08, *p* = 0.233).

(5) In the imaging subgroup (*n* = 218), the predictive value of the variables (i.e., normalized FDG uptake of 68 regions, global amyloid load (CL), and, for reference, 11 CERAD-NAB subtest scores) was significant only for semantic fluency (HR = 0.71 [0.63–0.80], *p* < 0.001, absolute Wald z statistic = 5.8), MMSE (HR = 0.93 [0.90—0.96], *p* < 0.001, absolute Wald z statistic = 4.4), naming (HR = 0.85 [0.79–0.91]), *p* < 0.05, absolute Wald z statistic = 4.3), figure drawing (HR = 0.86 [0.80–0.93], *p* < 0.05, absolute Wald z statistic = 4.0), and FDG uptake of the left inferior frontal gyrus, pars opercularis (HR = 0.68 [0.55–0.84], absolute Wald z statistic = 3.5; single-predictor Cox proportional hazard models, *p* < 0.05, all Bonferroni-corrected for 80 tests). Global amyloid load (CL) was not associated with mortality risk (HR = 0.93 [0.78—1.1], *p* = 0.406, absolute Wald z statistic = 0.8). Furthermore, we tested the predictive value of FDG uptake extracted from the Landau meta-ROI [15] and found that this was not associated with mortality risk (HR = 0.32 [0.06—1.66], *p* = 0.174, absolute Wald z statistic = 1.4).
(6) Again in the imaging subgroup, the predictive accuracy of the reference model (prediction by semantic fluency + age) was improved by additional inclusion of normalized FDG uptake in right and left caudal ACC, left inferior frontal gyrus, pars opercularis, right precentral, right and left superior frontal cortex (uncorrected *p* < 0.05, Fig. 4), but not global amyloid load (chisq = 1.77, df = 1, *p* = 0.183). In detail, greatest improvement was achieved by additional inclusion of right caudal ACC FDG uptake; the logrank test score changed from 37.4 to 48.8 (c = 0.69 to 0.70; chi^2^ = 10.6, df = 1, uncorrected *p* = 0.001, Bonferroni-corrected p for 69 tests = 0.078).

## Discussion

### Main findings

Analyses of cognitive variables showed that semantic fluency performance significantly predicted survival in memory clinic patients, i.e., mortality risk was approx. 40% higher per unit decrease of the age-, sex-, and education-adjusted Z score. Moreover, semantic fluency performance was the best predictor among CERAD-NAB subtests and, together with age, achieved a concordance of 67%; and it remained a significant predictor when amyloid status or global cognitive impairment were controlled for. A multivariable model confirmed that semantic fluency was the best (and only significant) predictor among CERAD-NAB subtests, and an elastic net multivariable model showed that semantic fluency retained predictive contribution also after accounting for correlated variables, which corroborated our finding. Longitudinal data showed highly significant mortality risk increase with greater semantic fluency decline.

We were able to demonstrate in a subgroup of patients with FDG PET and amyloid PET that worse semantic fluency correlated with decreased regional glucose metabolism of left-hemispheric temporal, frontal, and parietal cortex, but was not associated with amyloid load. Glucose metabolism of anterior cortical regions was itself a significant predictor and slightly improved prediction accuracy by semantic fluency.

Aformentioned findings considerably widened those of previous studies: semantic fluency has been shown to predict survival in healthy elderly [7] and in patients with clinical AD [3], but the latter study did not employ a biomarker-based diagnosis of AD and therefore it is unclear whether the finding holds true for biomarker-confirmed AD. We showed for the first time that semantic fluency is predictive for survival in memory clinic patients independently of their amyloid status (and hence the presence or absence of AD as defined in [13]) and, moreover, that longitudinal decline of semantic fluency is also a significant predictor.

Semantic fluency showed an added value over age, which is an established and obvious predictor of survival, even in patients with neurodegenerative diseases. Sex, however, was no predictor – in contrast to other studies’ findings (e.g., [16]) – and was hence not adjusted for in statistical models.

We regard worse semantic fluency to reflect greater disease severity. It might be a better disease severity marker in memory clinic patients than the established MMSE, which in the current work achieved only a concordance of 64% compared to 67% achieved by semantic fluency, in models adjusted for age. Noteworthy, the semantic fluency test of the CERAD-NAB is an extremely fast and simple test (even compared to the MMSE), which takes only one minute to be performed.

Semantic fluency is generally considered to rely on multiple cognitive processes, among them the critical steps of semantic memory retrieval and executive functions, the latter comprising at least the subprocesses of shifting (between mental search clusters), updating (a working memory component that keeps track of already produced items), and inhibition (of inappropriate responses). Notably, executive functions have traditionally been linked to the frontal cortex. As it is a language-based tests, it mainly examines left-hemispheric integrity. This is corroborated by its association with regional normalized glucose metabolism of the left frontal cortex.

The reason why executive functions and anterior cortical regions (frontal and ACC) are associated with mortality risk remains to be elucidated. The observed significant predictive value for survival of semantic fluency in particular might also in part be attributable to its psychometric properties: Compared with the other cognitive measures included in the study, semantic fluency may offer a wider range of possible scores and be less susceptible to floor or ceiling effects, potentially enhancing its sensitivity to clinically meaningful differences. However, this association has previously been demonstrated in a memory clinic cohort with the clinical diagnosis AD [17]. Similarly, using a large data set of patients from the National Alzheimer's Coordinating Center (NACC), a recent study [18] identified nine features predictive of survival in dementia that included also the TMT-B, a test commonly also associated with executive functioning. As executive dysfunction is a characteristic feature of the behavioral variant of frontotemporal dementia (bvFTD) [6], and bvFTD is associated with higher mortality risk [4, 5], it might be speculated that it is this group that drives the association that we observed. However, we found that amyloid status had virtually no effect on the predictive value of semantic fluency. Similarly, Pillai et al. [19] showed that worse working memory, a cognitive function also associated with the frontal lobe, predicted faster decline in AD.

Rhodius-Meester et al. [17] speculated that the reason for an association with mortality might be that subjects with executive dysfunction are at greater risk of dependency, increasing the risk of complications. Executive dysfunction may also lead to apathy, social withdrawal, and reduced self-initiated activity [9]. These might, via prolonged immobility or bed-rest, adversely affect the musculosceletal and cardiovascular systems [10, 11] and increase patients’ mortality risk [20]. Alternatively, it might be the case that a yet unknown health-related variable is independently associated with, on one side, executive dysfunction and anterior cortical neuronal injury, and mortality risk on the other. Candidate variables might be smoking, alcohol consumption, or diabetes, which have been linked to frontal lobe dysfunction [21, 22].

Apart from lifestyle factors (e.g., smoking, physical activity, and alcohol consumption), comorbidities, like endocrine-metabolic disorders, circulatory system diseases, and chronic kidney disease might affect survival in AD [23]. By contrast, Rhodius-Meester et al. [17] found no association of survival with comorbidities in AD. The current work might encourage further research to not only replicate our findings, but also test whether combinations with other candidate predictors help to explain current observations and improve prognostic models. Our view that cognitive test performance might still contribute to survival prediction when considering other predictors is supported by a recent study which found that dementia-related predictors such as specific neuropsychological tests were good predictors and minimally affected by other age-related causes of death, e.g., stroke and cardiovascular conditions [18].

Amyloid load had no predictive value: in amyloid-positive (A +) patients, amyloid load has presumably reached (individual) plateaus. In amyloid-negative (A-) patients, amyloid load naturally shows a floor effect. In the total group, amyloid load had no predictive value and amyloid status was not significant in an age-adjusted Cox model that included semantic fluency as, presumably, the A- group is highly heterogeneous and contains persons with FTLD (and short survival, [4, 5]), but also persons without any neurodegenerative disease, e.g., persons with cognitive complaints due to depression, a frequent differential diagnosis in memory clinics. Moreover, an FDG PET summary metric of the AD-typical posterior hypometabolism (FDG uptake in the Landau metaROI) also had no predictive value for survival in our cohort.

### Limitations

Due to the retrospective nature of the study, data acquisition not always followed a strict protocol as in a clinical trial. Hence there are missing data, and cases with missing data were excluded, thus restricting the current sample size. However, the study design still enabled us to analyze data from a considerable number of memory clinic patients. A further limitation is the unknown generalizability of our findings. However, similar findings from previous studies [3, 7], notably also in the general elderly population [7], might suggest generalizability to an elderly population. In our study, semantic fluency was the only measure of executive function whose predictive value was tested. It cannot be ruled out that other executive measures might exhibit even greater predictive value. Future studies could incorporate measures of behavioural change, which may provide additional prognostic information.

## Conclusions

Survival of memory clinic patients can be predicted by semantic fluency, independently from amyloid status and global cognitive impairment. Anterior cortical glucose metabolism is itself a significant predictor of survival and slightly improves prediction by semantic fluency.

## Supplementary Information

Supplementary Material 1.

## Data Availability

The data that support the findings of this study are available on request from the corresponding author. The data are not publicly available as they contain information that could compromise the privacy of research participants.

## Abbreviations

A+: Amyloid-Positive
A-: Amyloid-Negative
AD: Alzheimer’s Disease
bvFTD: Behavioral Variant Frontotemporal Dementia
CERAD-NAB: Neuropsychological Assessment Battery of the Consortium to Establish a Registry for Alzheimer’s disease
CL: Centiloids
CT: Computed Tomography
FDG: Fluorodeoxyglucose
FTLD: Frontotemporal Lobar Degeneration
GAAIN: Global Alzheimer’s Association Interactive Network
HR: Hazard Ratio
IFG: Inferior Frontal Gyrus
MBq: Megabecquerel
MMSE: Mini-Mental Status Examination
PET: Positron Emission Tomography
PiB: Pittsburgh Compound B
ROI: Regions-of-Interest
SPM: Statistical Parametric Mapping
