# Correction: Semantic fluency predicts survival of memory clinic patients

**DOI:** 10.1186/s13195-026-02199-x

**Published:** 2026-10-06

**Authors:** Lars Frings, Joachim Brumberg, Philipp T. Meyer

**Affiliations:** Department of Nuclear Medicine, Medical Center - Faculty of Medicine, University of Freiburg, Hugstetter Str. 55, Freiburg, 79106 Germany

**Correction: Alz Res Therapy 18, 181 (2026)**

**https://doi.org/10.1186/s13195-026-02152-y**

Following publication of the original article, it was noticed that Fig. 2 was incorrectly displayed due to a production error, resulting in the four survival curves being shifted to the top left. The incorrect and correct figure are shown below

The incorrect figure 2

**Figure:**
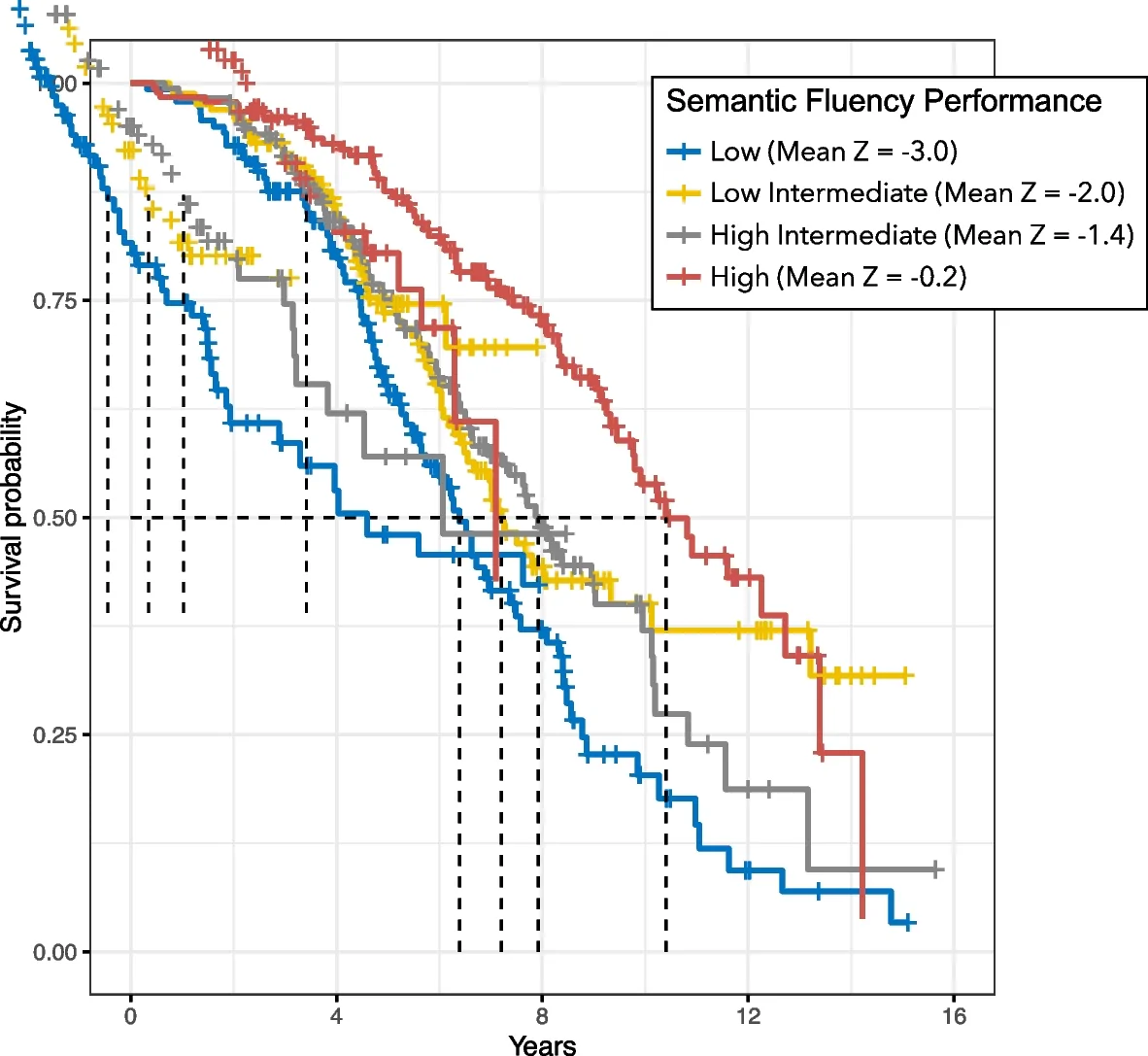

The correct figure 2

**Figure:**
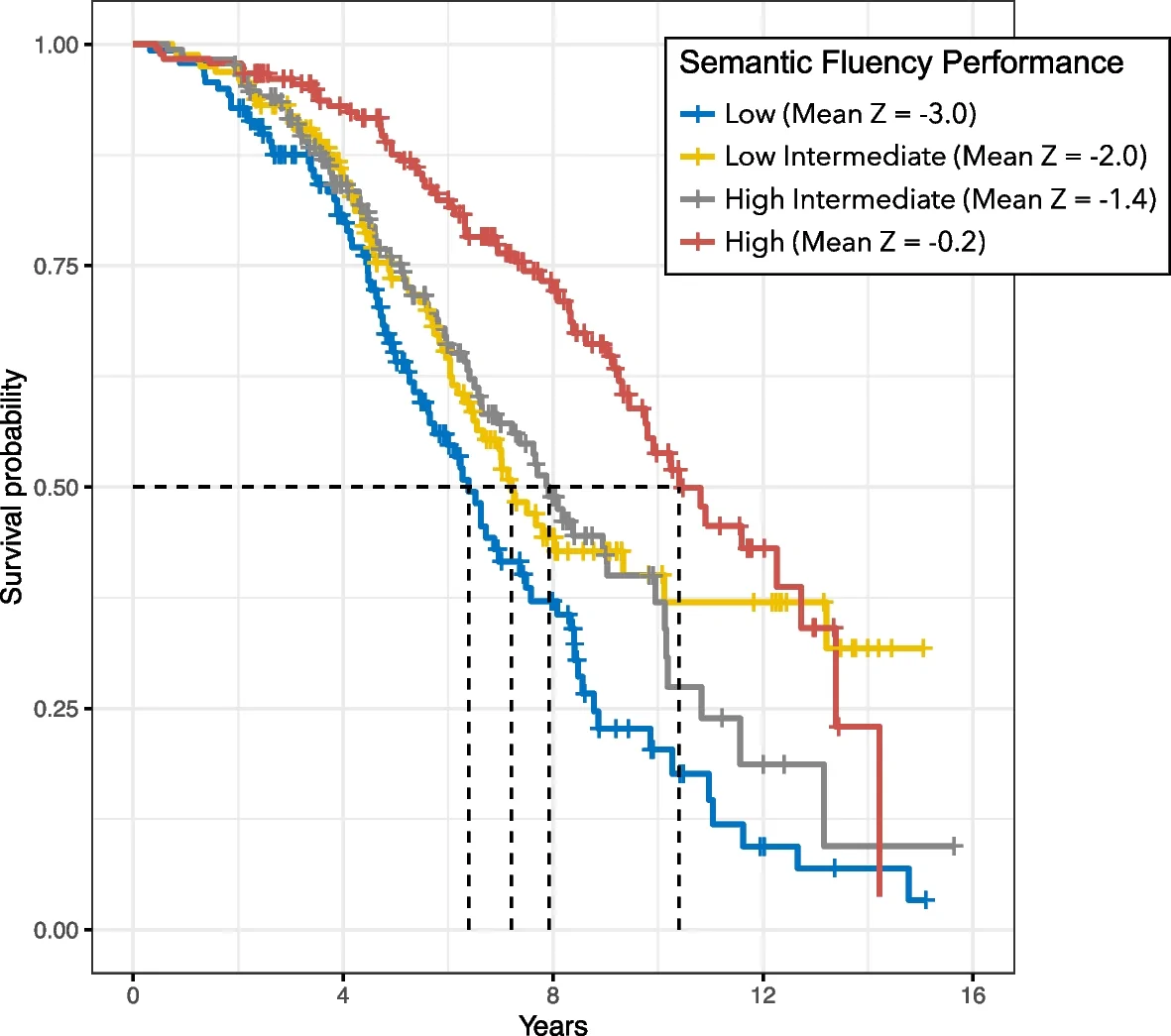

The original article has been updated.

