# Supplementary material for: Semantic fluency predicts survival of memory clinic patients
Source: Alzheimers Res Ther. 2026 Aug 7;18:181. doi: 10.1186/s13195-026-02152-y (PMC13452100; doi:10.1186/s13195-026-02152-y)
Supplement: Supplementary file 1 [file 13195_2026_2152_MOESM1_ESM.docx]

SUPPLEMENT

**Supplementary Table 1.** Overview of statistical models and significant results

|  | Models | N | Variable | Test statistic | P value |
| --- | --- | --- | --- | --- | --- |
| 1 | Single-predictor, age-adjusted Cox: coxph(Surv(time, event) ~ age + (one of 11 CERAD-NAB variables) | 583 | semantic fluency | HR = 0.71 [0.63-0.80] | < 0.001 (Bonferroni-corrected (B-corr) for 11 tests)  (see Suppl. Table 2 for details) |
|  | Analysis of deviance: reference model (coxph(Surv(time, event) ~ age) vs extended model (coxph(Surv(time, event) ~ age + semantic fluency) | 583 | + semantic fluency | Chi^2^ = 34.6 | < 0.001 |
|  | Multivariable Cox: coxph(Surv(time, event) ~ age + 11 CERAD-NAB variables | 583 | semantic fluency | HR = 0.73 [0.63 – 0.84] | < 0.001  (see Suppl. Table 2 for details) |
| 2 | Single-predictor, age-adjusted Cox: coxph(Surv(time, event)  ~ age + semantic fluency + MMSE  ~ age + semantic fluency + amyloid status | 583  218 | semantic fluency  MMSE  semantic fluency  amyloid status | HR = 0.75 [0.66 – 0.85]  HR = 0.96 [0.93 – 0.9997]  HR = 0.70 [0.58 – 0.84]  HR = 0.94 [0.65 – 1.37] | < 0.001  < 0.05  < 0.001  0.761 |
| 3 | Single-predictor, age-adjusted Cox: coxph(Surv(time, event) ~ age + change of semantic fluency | 163 | Change of semantic fluency | HR = 0.66 [0.53 - 0.83] | < 0.001 |
| 4 | Correlation of semantic fluency with 69 imaging variables: cor.test(.x, semantic fluency) | 218 | L ITG  L MTG  L MFG caudal  L IFG p.o.  L MFG rostral  L inferior parietal  L supramarginal | r = 0.35  r =0.33  r =0.31  r =0.29  r =0.28  r =0.27  r =0.24 | < 0.001 (B-corr for 69 tests)  < 0.001 (B-corr for 69 tests)  < 0.001 (B-corr for 69 tests)  < 0.05 (B-corr for 69 tests)  < 0.05 (B-corr for 69 tests)  < 0.05 (B-corr for 69 tests)  < 0.05 (B-corr for 69 tests) |
| 5 | Series of single-predictor Cox models (age-adj.) in 69 imaging and 11 CERAD-NAB variables | 218 | semantic fluency  MMSE  naming  figure drawing  FDG L IFG p.opercularis | HR = 0.71 [0.63 – 0.80]  HR = 0.93 [0.90 - 0.96]  HR = 0.85 [0.79 – 0.91]  HR = 0.86 [0.80 – 0.93]  HR = 0.68 [0.55 – 0.84] | < 0.001 (B-corr for 80 tests)  < 0.001 (B-corr for 80 tests)  < 0.05 (B-corr for 80 tests)  < 0.05 (B-corr for 80 tests)  < 0.05 (B-corr for 80 tests) |
| 6 | Analysis of deviance (ref model: coxph(Surv(time, event) ~ semantic fluency + age) vs extended models with one of 69 imaging vars each | 218 | + FDG R caud ACC | Chi^2^ = 10.6 | 0.078 (B-corr for 69 tests) |

**Supplementary Table 2.** Detailed results of the single-predictor Cox models (age-adjusted) and the multivariable Cox proportional hazard model (age-adjusted).

|  | **HR** | **lower .95** | **upper .95** | **p** | **HR** | **lower .95** | **upper .95** | **p** |
| --- | --- | --- | --- | --- | --- | --- | --- | --- |
|  | **single-predictor Cox models (age-adjusted)** | | | | **multivariable Cox model (age-adjusted)** | | | |
| **Semantic Fluency** | 0.71 | 0.63 | 0.80 | 7.581385e-09 | 0.73 | 0.63 | 0.84 | 1.60e-05 |
| **Wordlist Intrusions** | 0.93 | 0.84 | 1.02 | 0.1388 | 0.91 | 0.82 | 1.01 | 0.0779 |
| **Wordlist Recognition** | 0.98 | 0.90 | 1.05 | 0.5243 | 1.09 | 0.98 | 1.21 | 0.1162 |
| **MMSE** | 0.93 | 0.90 | 0.96 | 8.714990e-06 | 0.96 | 0.91 | 1.01 | 0.1353 |
| **Figure Drawing** | 0.86 | 0.80 | 0.93 | 7.669804e-05 | 0.92 | 0.83 | 1.03 | 0.1370 |
| **Naming** | 0.85 | 0.79 | 0.91 | 2.053972e-05 | 0.95 | 0.86 | 1.04 | 0.2426 |
| **Wordlist Recall** | 0.94 | 0.86 | 1.03 | 0.1779 | 1.10 | 0.84 | 1.43 | 0.4910 |
| **Wordlist Recall (proportion)** | 0.97 | 0.91 | 1.04 | 0.4045 | 0.96 | 0.85 | 1.10 | 0.5616 |
| **Figure Recall (proportion)** | 0.97 | 0.89 | 1.06 | 0.5226 | 1.05 | 0.87 | 1.26 | 0.6036 |
| **Wordlist Learning** | 0.91 | 0.84 | 0.99 | 0.0233 | 1.00 | 0.86 | 1.18 | 0.9608 |
| **Figure Recall** | 0.92 | 0.85 | 0.99989 | 0.0497 | 1.00 | 0.81 | 1.23 | 0.9910 |

**Supplementary Table 3.** Predictive value for survival (single-predictor Cox models, age-adjusted) of annual performance change of CERAD subtest scores (based on a linear mixed effects model with random intercepts and random slopes for follow-up time).

|  | **Number of patients with multiple assessments (proportion of the total number of 583 analyzed patients in %)** | **Number of assessments** | **decline per year (slope in Z scores adjusted for age, sex, and education; M ± S.D.)** | **HR** | **lower .95** | **upper .95** | **p** |
| --- | --- | --- | --- | --- | --- | --- | --- |
|  |  |  |  |  | | | |
| **Semantic Fluency** | 163 (28%) | 402 | -0.12 ± 0.05 | 0.66 | 0.53 | 0.83 | p < 0.001 |
| **MMSE** | 202 (35%) | 526 | -1.51 ± 1.37 [slope in MMSE points per year] | 0.62 | 0.52 | 0.76 | p < 0.001 |
| **Figure Drawing** | 155 (27%) | 373 | -0.13 ± 0.05 | 0.82 | 0.66 | 1.02 | p = 0.076 |
| **Wordlist Learning** | 154 (26%) | 370 | -0.17 ± 0.05 | 0.85 | 0.69 | 1.04 | p = 0.115 |
| **Naming** | 167 (29%) | 403 | -0.14 ± 0.12 | 0.84 | 0.67 | 1.05 | p = 0.116 |
| **Wordlist Recognition** | 154 (26%) | 369 | -0.12 ± 0.03 | 0.98 | 0.80 | 1.20 | p = 0.827 |
| **Wordlist Recall** | 154 (26%) | 370 | -0.13 ± 0.06 | 0.88 | 0.71 | 1.09 | p = 0.246 |
| **Wordlist Recall (proportion)** | 154 (26%) | 370 | -0.06 ± 0.04 | 0.98 | 0.79 | 1.21 | p = 0.822 |
| **Wordlist Intrusions** | 153 (26%) | 368 | * | * | * | * | * |
| **Figure Recall** | 153 (26%) | 367 | -0.09 ± 0.01 | 0.88 | 0.70 | 1.10 | p = 0.257 |
| **Figure Recall (proportion)** | 153 (26%) | 367 | * | * | * | * | * |

*Individual mixed-effects slopes could not be estimated for this cognitive measure because the random-slope variance was estimated as zero (singular fit), indicating insufficient evidence for between-subject variability in longitudinal change. The predictive value was hence not tested.
